# Effort, symptom validity testing, performance validity testing and traumatic brain injury

**DOI:** 10.3109/02699052.2014.947627

**Published:** 2014-09-12

**Authors:** Erin D. Bigler

**Affiliations:** ^a^ Department of Psychology; ^b^ Neuroscience Center; ^c^ Magnetic Resonance Imaging Research Facility, Brigham Young University Provo, UTUSA; ^d^ Department of Psychiatry; ^e^ The Brain Institute of Utah, University of Utah Salt Lake City, UTUSA

**Keywords:** Effort testing, neuropsychological assessment, performance validity testing, symptom validity testing, traumatic brain injury

## Abstract

*Background*: To understand the neurocognitive effects of brain injury, valid neuropsychological test findings are paramount.

*Review*: This review examines the research on what has been referred to a symptom validity testing (SVT). Above a designated cut-score signifies a ‘passing’ SVT performance which is likely the best indicator of valid neuropsychological test findings. Likewise, substantially below cut-point performance that nears chance or is at chance signifies invalid test performance. Significantly below chance is the *sine qua non* neuropsychological indicator for malingering. However, the interpretative problems with SVT performance below the cut-point yet far above chance are substantial, as pointed out in this review. This intermediate, border-zone performance on SVT measures is where substantial interpretative challenges exist. Case studies are used to highlight the many areas where additional research is needed. Historical perspectives are reviewed along with the neurobiology of effort. Reasons why performance validity testing (PVT) may be better than the SVT term are reviewed.

*Conclusions*: Advances in neuroimaging techniques may be key in better understanding the meaning of border zone SVT failure. The review demonstrates the problems with rigidity in interpretation with established cut-scores. A better understanding of how certain types of neurological, neuropsychiatric and/or even test conditions may affect SVT performance is needed.

## Introduction

How should valid neuropsychological test performance be determined in the individual with traumatic brain injury (TBI), especially those who have sustained a mild TBI (mTBI)? The commonness of mTBI with annual incidence rates conservatively estimated to be well over 2 million cases per year [1], likely means that mTBI represents one of the most common forms of injury evaluated by neuropsychologists [2]. Validity of neuropsychological test findings has become a critical issue in the assessment of neurocognitive and neurobehavioural sequelae, where hundreds of *Brain Injury* articles and published abstracts include statements about or information on what has been referred to as symptom validity testing (SVT). Currently, SVT research is a dominant theme in the field of neuropsychological assessment [3, 4]. As will be discussed in this review, the SVT term may not be the best to use in this context, but for brevity this common acronym will be retained because of its entrenched use in current neuropsychological literature [4].

Over the past decade, there has been wide acceptance for the use of SVT measures in clinical neuropsychological assessment as well as research [5]. Prior to the current era of specific SVT studies, valid neuropsychological test performance was always a concern but essentially left to clinical judgement based on observed test behaviours like patterns of deficit performance that did not fit clinical history or presentation along with obvious signs of lack of engagement in the testing process. In an attempt to bring greater objectivity in reporting test performance validity, about two decades ago externally administered SVT measures began to be routinely applied during neuropsychological assessment batteries, especially in the evaluation of mTBI [6].

SVT tasks may be divided into free-standing measures independently administered from the other neuropsychological tests, but used to infer validity, from devised SVT criteria extracted from embedded tests within a standardized battery of neuropsychological tests administered or a combination of these two approaches. Although recommendations from professional neuropsychological organizations endorse the use of external SVT measures [5, 7, 8], there is no agreement on which ones should be used for which condition, the timing of when SVT measures should be administered in the context of other tests, how many and what guidelines should be used for interpretation, to identify but a few of the important unresolved issues.

The most common SVT research study and most widely published articles are based on externally administered SVT measures that use an easy to perform task often within a forced-choice (FC) paradigm, typically involving some aspect of recognition memory [9]. The majority of the FC SVT measures in use are commercial products and this review is not about which may be best under what circumstances or a review of the assessment utility of individual SVTs. In that sense, this review will avoid specific mention of commercially available SVTs, will remain neutral on the purported test qualities of particular SVT measures and comment mostly on the SVT paradigm as it relates to inferences about validity.

Embedded SVT measures may capitalize on inherent features of the task, some of which permit utilizing a FC paradigm as well or the ease of some items, especially at the beginning of a measure. For example, even patients with severe TBI are routinely capable of repeating back a few digits in forward and reverse directions and, therefore, the individual who has sustained a mTBI that cannot perform even simple digit repetition is displaying invalid test performance. Most SVT measures, regardless of whether external or embedded, utilize a cut-score approach where performance above a certain cut-score reflects a ‘pass’ or ‘valid’ performance and ‘failure’ occurs with below cut-point performance, thereby inferring ‘invalid’ test findings. The concept of a SVT ‘pass’ seemed like a simple, quick unbiased way to comment on validity and, in fact, numerous studies support such a conclusion.

Truly invalid test performance also could be concluded from SVT performance. For example, near chance SVT scores unequivocally merit the ‘invalid’ moniker. Within the FC paradigm, near chance performance occurs with random responding or guessing, hardly an indication an examinee was trying to perform at their best. Below chance performance clearly implies intentionality and knowingly falsifying the answer. These types of ‘failed’ SVT performances provide the clinician/researcher with definitive and objective indicia for invalid neuropsychological test findings. Presence of such a pattern requires no further discussion in this review, especially SVT performance substantially below chance because this represents the psychometric *sine qua non* indicator for malingering (knowing the correct answer and intentionally identifying the incorrect).

However, there is an intermediate group, technically a SVT failure, characterized by below SVT cut-score performance but substantially above chance [10]. This represents a common observation in the neuropsychological assessment of mTBI, both paediatric and adult [11, 12]. Does this in-between, border zone SVT performance truly reflect invalid performance or do SVT measures tap cognitive or behavioural dimensions of test performance that require additional consideration?

The size of this group with substantially above chance yet below SVT cut-score performance is not trivial. In the various studies referenced in this review, where appropriate data were presented to determine this type of failure rate, it may be upwards of 20% of the total sample of brain injured patients [13]. However, the very nature of how cut-scores are derived calls into question why such a performance would automatically indicate invalid performance. As pointed out by Dwyer (1996), cut-scores (a) always entail judgement, (b) inherently result in some misclassification, (c) impose artificial ‘pass/fail’ dichotomies and (d) no ‘true’ cut scores exist [14]. The implications of these problems is easily grasped if in a 50-item FC task the cut-score is set at 90% accurate; in other words a score of 45 is needed to pass. So what does a score of 44 mean— it is below the cut-point but does this really mean invalid? Even a score of 40 still indicates 80% of the answers were correct. Does this intermediate range always mean ‘invalid’ performance just because the SVT score is below the cut-point?

This review focuses on the limitations of SVT research findings thus far and the questions that need answered for this intermediate, border zone ‘failed’ group of SVT performers with a history of TBI. Interpretation of any neuropsychological test requires operational definitions of the terms used to describe test findings. This certainly applies to SVT interpretation which means some historical overview of SVT terminology and potential operational definitions is needed.

## What is effort?

Early writings involving SVT studies introduced the term ‘effort’ to describe what was being measured by the SVT task. Intuitively the ease of the SVT measure has been interpreted as tapping some index of *effort*. In fact, use of the effort term for a while became synonymous with SVT, often used in the title of the study [15, 16] and is still being used to describe SVT studies. So what is effort?

This question has been asked before [10, 17]. On the surface, this seems straightforward. Effort has to be involved in test taking and must in some way tap the level of cognitive and behavioural engagement in a task. If not putting forth good effort to perform at their best level of ability, indeed their most accurate ability level, how could assessment ever hope to evaluate an individual’s ability to function? Instructionally, something to this effect is asked of every examinee prior to administration of any standardized neuropsychological assessment [18] when admonished to ‘do their best’, to give their ‘best effort’.

**Figure 1.**
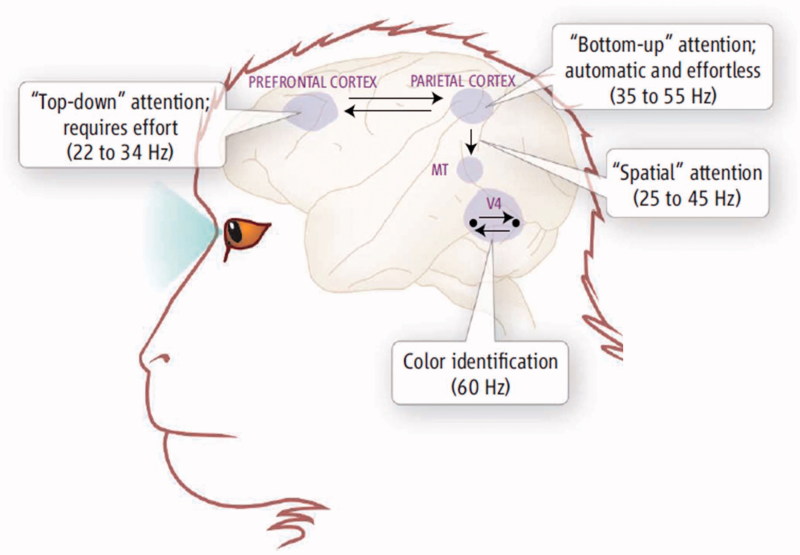
As shown in this illustration from Knight [19], only the ‘Bottom-up’ attention network is effortless and automatic. ‘Top-down’ attention, memory or visual processing all requires effort. The image is based on electrophysiological studies of the mammalian brain, as referenced by the oscillatory findings given as hertz (Hz) frequencies. Used with permission from Science.

From a basic neuroscience perspective, effort has been examined in a variety of ways by manipulating several stimulus and task parameters, most often within working memory (attention) and short-term retention paradigms. Figure 1 is from Knight [19], wherein the bottom-up vs. top-down attention network dichotomy is used to show that only bottom-up, primary sensory stimulation engages the cortex in an otherwise ‘effortless’ manner. All other types of cognitive processes require effort, no matter how insignificant or transitory the task may be. ‘Bottom-up’ stimuli, especially threat stimuli, immediately engage attentional networks and are, therefore, ‘effortless’. However, all other aspects of attention require cognitive effort and a central executive as borne out when functional MRI (fMRI) studies are done using simple FC or SVT measures [20–22]. These studies all show that SVT measures engage the expected language, memory, attentional and executive functioning networks necessary to perform the FC SVT task. Interesting to note, studies actually show greater and different activation when the subject is asked to malinger, suggesting that the neurobiology of malingering may have a signature fMRI activation profile [23, 24].

In cognitive neuroscience research paradigms, cognitive effort may be operationally defined by task complexity and experimentally manipulated as an independent variable. Such is not the case with SVT measures that have a fixed-battery approach, although several will vary between so-called ‘difficult’ and ‘easy’ items. Since all SVT paradigms would tap top-down cognitive processes, none, no matter how easy, could be considered effortless. Are there potential neuropathological factors that relate to ‘effort’ in cognitive processing? Do they influence performance and relate to validity of test findings, especially in TBI?

## SVT, malingering and the nature of deception

Contemporaneous with emergence of SVT and the ‘effort’ terminology was equating failed SVT performance with malingering. By DSM-5 standards [25] malingering is defined as ‘… the intentional reporting of symptoms for personal gain’ in contrast to factitious disorder that ‘… requires the absence of obvious rewards’ but both involve ‘… providing false information or behaving deceptively’ (p. 326). Malingering requires intentionality and numerous SVT publications loosely use the ‘malingering’ term in association with SVT failure, even though the only statistical proof of intentionality is when SVT performance is below chance. So what role does deception plays in SVT performance?

In their textbook titled *Malingering and Illness Deception*, Halligan et al. [26] begin their edited treatise with an overview of the commonness of deception at all levels of human behaviour as well as throughout the animal kingdom. Indeed, even the evolutionary basis to human deception has been written about [27, 28]. The crux of the problem with deception for medicine and psychology is the subjectivity of symptom reporting, since diagnosis relies, in part, on the credibility of symptoms. The complexity that surrounds subjective symptom reporting becomes exponentially amplified when issues of intentionality and motivation, conscious or non-conscious, enter the equation [29, 30]. So what does one conclude about a clinical presentation of a patient wherein there is no medical explanation or supporting finding for a specific disorder—the so-called ‘medically unexplained symptoms’ or MUS [31]? MUS patients complain of cognitive impairments [32] and, in fact, a community prevalence study of cognitive impairment found ∼12.5% to have MUS [33]. So if a MUS patient ‘passes’ SVT measures but has cognitive impairments on neuropsychological testing, are their cognitive problems genuine (valid)? What does it mean to pass SVT measures in those with presumed functional or psychogenic disorder? Kemp et al. [34] examined a sample of 43 non-litigating individuals all assessed with MUS, well characterized as having no medically diagnosable condition. None actually performed at or below chance level on the various SVT measures administered, but 11% ‘failed’ SVT tasks. Does this mean that those individuals with MUS who ‘pass’ SVT measures but exhibit neuropsychological impairment have valid test findings and a genuine cognitive disorder? Or, does this mean that even a ‘passed’ SVT finding does not necessary mean a bona fide cognitive impairment?

This issue is very important in understanding TBI, especially mTBI, because of the role of so-called functional predisposing, pre-injury mental health issues in TBI outcome [35] as well as what has been referred to as central sensitization where symptoms may not fit the clinical history [36]. Does under-performance on SVT measures tap what is referred to as ‘illness belief’ [37] and relate to psychogenic features related to outcome [35]? Is diminished SVT performance, whether intermediate or at chance, tapping some aspect of illness behaviour? In a large 1-year prospective sample (*n* = 1144) of neurology out-patients, Sharpe et al. [38] showed that illness beliefs and financial benefits predicted 1-year outcome. Although outcome prediction was multi-factorial and did not use specific SVT measures, illness belief and secondary gain predicted 13% of the variance in outcome [39, 40]. In neuropsychological assessment, once issues of litigation and secondary gain enter into the clinical picture then the potential explanatory power of failed SVT performance increases, even within the intermediate category of above chance, but below the cut-score performance [41]. Accordingly, performance on SVT measures tap test-taking behaviours that have relevance to issues of secondary gain, deception and illness behaviour. Nevertheless, illness behaviour in the form of what DSM-5 now describes as Somatic Symptom Disorder constitutes a bona fide psychiatric disorder. Is this intermediate, border zone range of SVT performance a reflection of illness behaviour? Can SVT measures for those who perform in this intermediate range be used to decipher what may or may not be illness behaviour?

## Historical perspective and nomenclature

At the mild end of the TBI spectrum, the debate on the validity of neurocognitive and neurobehavioural symptoms resulting from a mild brain injury is not new. Although Courville’s [42] text *Commotio Cerebri* argued for a biological basis to concussion, he acknowledged the absence of objective findings in concussion supported the view by many

… that the symptoms so often complained of consequent to an episode of concussion constitutes nothing more than a psychogenic disorder inherent in the patient’s deficient personality (traumatic neurosis), if he is not actually guilty of an *effort* to secure gain by fraud (malingering) (p. 3).

More than 60 years ago, Courville’s [42] use of the word ‘effort’, which would portend the zeitgeist around validity assessment in psychological and neuropsychological testing in TBI, began with FC methods for the detection of malingering [43–45]. As implied in the above Courville statement, effort may reflect malevolent aims where deficits are intentionally exaggerated, under-performed or test performance feigned, which has been a long-held view as the basis for many who have persisting problems from a mTBI [46].

Is their intrinsic motivation to perform at maximal effort at all times [47]? The assumption in psychological assessment has always been that the demand characteristics and social context of the examinee–examiner test environment provide motivation to adequately perform [48]. Indeed, for the majority undergoing testing the demand characteristics of the assessment environment results in passing SVT measures. For example, in an active and veteran military study of 214 individuals, many with a history of mTBI, McCormick et al. [49] found that 75% performed above the SVT cut-point used in that study. This implies that in the majority the demand characteristics of the test environment provide sufficient motivation for valid performance, by SVT cut-point standards. However, sufficient motivation is not necessarily the same as optimal performance on neuropsychological measures. Indeed, every neuropsychological measure has its own test–re-test variability which may vary considerably across different time points [50]. So passing a SVT may signify a ‘valid’ performance but may not address whatsoever the range of test performance or whether performance was optimal.

Traditional neuropsychological testing is not performed with implicit incentives other than ‘try your best’. In the traditional sense incentive is not manipulated like an independent variable in a research study on cognitive effort. As such, motivation is not directly assessed unless inferred from SVT measures. Financial incentives have the potential to influence intrinsic motivation [51], so how should SVT measures deal with secondary gain? Do illness behaviours or threats to perceived health status, so-called diagnosis threat, influence motivation to perform [52–54]? Probably, but what if core features of the neurological or neuropsychiatric disorder influences motivation? Is there an interactive effect between illness behaviour, neurological/neuropsychiatric condition and SVT? There is an entire field within cognitive neuroscience that explores issues of motivation, drive, response-cost and brain function [55, 56], as well as how clinical disorders affect motivation [57]—how do these factors influence SVT performance? The case study presented in the next section highlights this interpretive dilemma.

A consistent argument made by SVT publishers and researchers is the ease of the FC tasks could not be simpler and therefore require ‘minimal or no effort’ to pass. SVT standardization samples include individuals with neurological impairment who make few to no errors under standard format. Even some convenience samples involving those with intellectual disabilities or otherwise severe neurological impairment readily pass SVT measures [58, 59], all of which supports a minimalist role of motivation to adequately perform. On the other hand, as will be shown in the TBI case that follows, neurological injury may affect motivational systems and presumably SVT performance. If the neurobiology and neuropathology of the injury may explain intermediate, border zone SVT performance, then concluding invalidity of neuropsychological test findings may not be an empirically supportable conclusion.

## A mTBI case study of below cut-score SVT findings with well-documented neuropathology: The prototype problem for the SVT researcher and clinician

**Figure 2.**
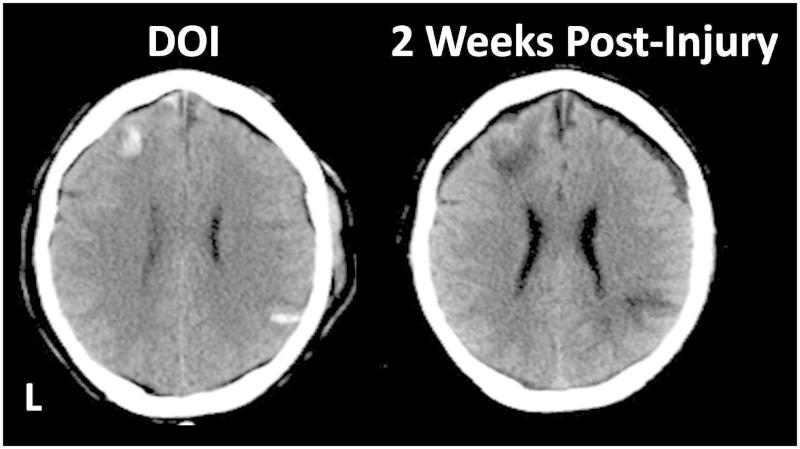
L = Left. The CT image on the left is from the day-of-injury (DOI) and shows focal intraparenchymal haemorrhage in the left frontal lobe with a contrecoup focal haemorrhagic lesion located posteriorly in the right parietal area. By 2 weeks post-injury, focal areas of decreased density deep within the frontal and parietal parenchyma have developed, reflecting damage substantially larger than the original haemorrhagic lesion.

This patient sustained a high impact front-end collision with immediate loss of consciousness (LOC) and an initial Glasgow Coma Scale score of 5. Multiple fractures were sustained, including orbitofacial and spinal column, internal injuries and CT demonstrated a distinct frontal haemorrhage associated with a right contrecoup parietal haemorrhage, as shown in Figure 2. Clearly evident (also see Figure 2), follow-up CT imaging demonstrated even larger parenchymal changes as a result of the original haemorrhagic lesions and brain injury (MRI studies were never done with this patient because of artifact from the craniofacial reconstruction). Behaviourally the family described a change in motivation characterized by apathy and lack of drive as well as reduced cognitive ability, characteristic of trauma-induced frontal lobe disorder [18]. Prior to sustaining the severe TBI, the individual shown in Figure 2 was college educated with executive level employment. By outcome standards the patient had good recovery (Wechsler Abbreviated Scale of Intelligence, Full Scale IQ = 112, Wechsler Memory Scale-III Memory Quotient = 108) and was able to do all levels of personal care and activities of daily living including resuming driving. However, are these scores invalid because of SVT failure? When seen for neuropsychological evaluation, he was 2-years post-injury and litigation was present, but settled out of court. As for SVT performance he scored right at the cut-point on one SVT measure but at only at a 78% correct level on another where the established cut-point is 85%. His Beck Depression Inventory-II score was 37. On mental status exam, he expressed that he ‘… doesn’t give a shit about things!’ Does frontal lobe damage, depression and lack of motivation influence SVT performance? Does parietal lobe damage influence attention and lack of task engagement that could lead to infrequent but some errors sufficient to score below the cut-point? Do the neuroimaging findings in this case support top-down disruption of the effort system? SVT researchers have provided few guidelines for what plausibly may be failed SVT performance due to neurogenic motivational and attentional problems in neurologic patients with unequivocally documented brain pathology such as this case; however, some studies are beginning to address these issues [60].

What would it mean in a patient with SVT ‘failure’, like that in Figure 2, if the neuropsychological test findings are reflexively interpreted as implicating invalid symptom endorsement and behavioural deficits post-injury? Obviously neuroimaging demonstrates structural damage to key regions associated with motivation and drive as well as attention. Does the SVT ‘failure’ in this case actually prove the point of poor engagement that would be expected from frontal lobe damage? Is the SVT then acting as a cognitive probe that actually detects what would be predicted from such a brain injury [61]? Why would this patient’s neuropsychological test performance be interpreted as invalid because of SVT failure in the intermediate, border zone range, especially since summary scores are all above average?

This case also points out a major limitation of commercially used SVT measure. Their standardization samples combine so-called ‘neurological’ patients of all types of aetiology within a single group (mixed acquired injury, stroke and dementia, for example). However, as pointed out by the case in Figure 2 there may indeed be variation in SVT performance by type of disorder and location of pathology [62] as well as type of neuropsychiatric disorder [63]. Researchers are showing that the inflexible cut score should be reconsidered [64, 65]. Most commercially available SVT measures treat the ‘brain damaged’ or ‘neurologically impaired’ group as a singular group assuming there is some unitary dysfunction that would accompany brain damage resulting from multiple aetiologies, without ever really testing this assumption.

## What modifies SVT and neuropsychological test performance?

**Figure 3.**
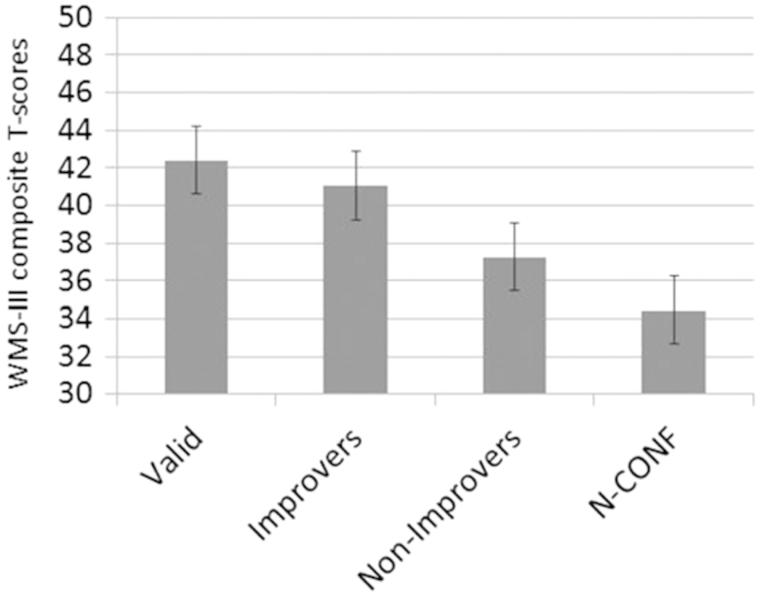
This illustration is from Suchy et al. [66] and demonstrates the dose–response relationship frequently reported between increasingly lower SVT performance and actual neuropsychological test findings [3]. Mean composite WMS-III T-score for individuals in the four groups were calclated: Valid = patients who produced above cut-score SVT performance during initial administration; Improvers = patients who initially produced non-valid SVT performance, but the repeat administration after confrontation yielded valid results; Non-improvers = patients who produced non-valid SVT performance initially and again after confrontation; N-CONF = patients who initially produced non-valid VSVT performance but were not confronted. Reproduced with permission from the author and Psychology Press/Taylor & Francis.

Changing examiner/examinee demands may indeed change performance or motivation to perform on neuropsychological tests, as shown by Suchy et al. [66], using a most resourceful archival research design involving clinical cases (*n* = 530) with documented multiple sclerosis (MS). Diagnosis was not in question as all patients had all been independently diagnosed with MS, none were in litigation and all were merely being evaluated for treatment planning or follow-up. Eleven per cent failed FC SVT measures. Interestingly, confronting those who initially exhibited below cut-score SVT scores resulted in 68% being able to raise their SVT scores into the ‘pass’ category, resulting in Wechsler Memory Scale (WMS-III Edition, Wechsler) performance equivalent to the group that originally passed (see Figure 3). Could this be a strategy employed by the clinician or researcher because confronting the patient with below cut-score SVT performance who then improves constitutes some sort of proof that the patient was not fully engaged in performing the task?

What about those MS patients who did not improve when challenged, how substantial is the effect on WMS-III performance when SVT is ‘failed’ below the cut point and does not change during the challenge? Figure 3 shows this comparison. First, what is evident in viewing this figure is the so-called dose–response relation between level of SVT performance and neuropsychological test performance, in this case WMS-III performance [3]. Passing on the first SVT administration resulted in the highest performance, with below cut-point performance related to lower WMS-III performance. However, regardless of whether the SVT was passed, *all* MS groups performed below the normative standard. Furthermore, the ‘Non-Improvers’—those who truly ‘failed’ the SVT measure—were just a 0.5 standard deviation (SD) below those who passed.

In a somewhat similar study, Keary et al. [67] examined patients with intractable epilepsy who were potential candidates for surgery with no know external incentive to malinger. In this cohort they examined three SVT performance findings: Valid (meaning at or above cut-score), Questionable (substantially above chance, but below the SVT cut-point) and Invalid (substantially below the cut point). On the WMS-III the valid group performed at ∼0.75 SD below the standardization sample, with the ‘questionable’ SVT group performing on average 1.0 SD below. The epilepsy group that failed performed 1.66 SD below the standardization sample or ∼0.9 SD worse than those who passed.

Ignoring whether valid or not, the neuropsychological test performance in these samples of independently diagnosed conditions that would be expected to reduce cognitive ability in fact show lower neuropsychological test performance regardless of whether the SVT was passed or not. While the magnitude of the difference may be influenced by factors such as motivation, test engagement, effort of whatever term might be appropriate, actual SVT performance was irrelevant in terms of the direction of impairment.

Furthermore, Keary et al. [67] observed that both working memory and intellectual level were related to SVT performance, stating the following:

… the cognitive profile should not automatically be judged invalid. Instead, clinicians will need to consider the possibility that lower performance … may be due to intellectual or working memory limitations (p. 321),

irrespective of SVT findings. These findings point to the limits of hard-and-fast rules of distinguishing valid versus invalid test performance based solely on a SVT cut score without, as Keary et al. [67] state, looking ‘… for convergence of findings’ (p. 321).

Locke et al. [13] examined patients for treatment determination, where the majority had sustained a TBI, none were in litigation, with ∼20% failing SVT measures. For the group that performed below the SVT cut-point, on the majority of neuropsychological measures, test performance was significantly below the group who passed, with effect size differences ranging from 0.21–1.37. While moderate-to-large effect size differences may be substantial enough to result in clinically important and distinguishable differences, small effect size differences may not [68]. In children, universal differences in test performance have not been observed for those who failed SVT in contrast to those who passed [62, 69]. Such findings challenge the argument that failed SVT performance collectively and always reflects invalid test performance across the board for all other neuropsychological measures. In fact, Perna and Loughan [69] state that ‘sub-optimal effort … may not predict poorer performance on a neuropsychological evaluation in children, as has been reported in other studies’ (p. 31). Nonetheless, substantial rates of SVT failure in paediatric mTBI have been reported [70–72].

The complexity of motivational factors is demonstrated by studies that use monetary incentives to improve performance [73–75]. In children, McCauley et al. [76, 77] have shown that performance on prospective memory tasks in those with mild/moderate TBI improved over baseline assessment with increased monetary incentive. What does this mean? Was the first assessment done under conventional terms of no incentive less than optimal or invalid?

Schizophrenia is often characterized by deficits in motivational systems [78] and SVT studies that have examined SVT failure rates show high levels of failure in those with schizophrenia [79–82]. Indeed major discussions in the schizophrenia literature centre on whether amotivational features of the disorder occur as a state vs. trait [83]. If motivation or drive is a state, then if sub-optimal performance is part of that state, even those with below cut-score but above chance SVT performance may be performing at their ‘typical’ level of task engagement.

Since motivation, intention and choice may be framed by neural structures that underlie drive including medial and orbitofrontal cortices and their relation with limbic and hypothalamic areas [84], SVT explanations in this indistinct category of below cut-score performance must deal with whether certain lesions or neuropathological states are associated with motivational changes that may influence SVT performance. If this is the case, then motivation becomes another term to operationalize and define in understanding SVT findings. Unfortunately, although the motivation term has great face value along with intuitive explanatory appeal, the term possesses many challenges to operationally define [85, 86].

All externally administered SVT measures have been standardized via samples of convenience. In clinic situations that have examined consecutive referrals, some find low SVT failure rates comparable to standardization samples, but others do not [13, 66], where in multi-specialty clinical services in large metropolitan centres up to 30% fail FC SVT measures [87, 88]. Substantially larger numbers have been reported in some military-related clinics where upwards of 60% SVT failure rates have been reported [89, 90]. As pointed out by Silver [35], there are many complexities to performance validity issues, especially in mTBI [91]. With such substantial SVT failure rates without either better operationalizing what below cut-score but above chance performance truly means will result in large numbers of assessment findings ostensibly discounted to a non-interpretable category. For the profession of clinical neuropsychology this is an untenable position to be in, if one concludes that these high levels of failure *only* mean invalid test performance.

## What is in a name?

There is more historical context for the operational definition challenges introduced above. The Rey Auditory Verbal Learning (RAVL) test [18], first published in 1941, also was the first neuropsychological measure that became connected with an external SVT, the Rey 15-item test and also the Dot-Counting Test (later referred to as the Rey Dot-counting test [92]). Rey recognized that understanding ‘motivation’ and ‘believability’ of test performance was needed. These measures were not FC-based, but rather deemed so basic and easy to perform that even patients with significant neurological impairment could adequately do the task with some minimal level of correctness [93]. As such, the Rey-15 and Dot-Counting measures were thought to tap an element of ‘motivation’ to participate in the RAVL task [94]. So, as early as 1964, Rey was writing about motivational factors and how to distinguish genuine vs. feigned memory impairment which continued to dominate much of the early writings of neuropsychological test validity prior to the 1990s [95].

Adding to what Rey started, by implementing a FC paradigm applied to the ease and simplicity of performing a task, the statistical improbability of poor test performance provided additional psychometric support for defining presence of implausible and malingered symptoms [96–99]. For example, implausible symptoms like someone with a history of mTBI who claimed to lose *all* memory of the previous day during sleep [100].

These techniques appear to do well in separating out examinees with truly improbable test findings and true malingerers. The conundrum as introduced by MUS conditions is that, in disorders considered to have a high degree of functional overlay, it would be expected that increased SVT failure occurs in higher rates where symptoms like fatigue and pain dominate, such as in chronic fatigue/fibromyalgia [101]. For example, Johnson-Greene et al. [102], in a study of patients with fibromyalgia, found a 37% SVT failure rate. SVT failure was associated with non-cognitive symptoms of pain, poor sleep and fatigue. Of course, these are the same kinds of symptoms that occur in mTBI, which then begs the question as to whether the SVT task itself is tapping some form of response bias that actually relates to the presenting problem/symptoms experienced by the patient. For example, returning to military sample, including those with mTBI reported earlier in this review, McCormick et al. [49] observed a 25% SVT failure where SVT performance was associated with depression and PTSD.

McGrath et al. [103] discuss ‘response bias’ and its influence on psychological test performance. Response bias is a well-studied, experimentally proven factor that may contribute to both accurate and false memory [104]. Response bias that occurs in schizophrenia [105] is likely a major factor associated with poor SVT performance [82]. However, in cognitive psychology and neuroscience, response bias is something proven by experimental manipulation of independent variables and test conditions by increasing or decreasing task demands, priming and stimulus complexity. Indeed, when these factors are attempted to be experimentally manipulated they demonstrate the arbitrariness of the SVT cut-point [106].

Much of the SVT research has been driven by forensic neuropsychology [107] where the ‘response bias’ is assumed because of compensation seeking and issues of secondary gain? In forensic samples often a comparison group is derived that is in litigation in contrast to a non-litigating sample. When greater SVT failure occurs in the litigating sample, a negative response bias is assumed, attributed to the motivation for secondary gain. As Merten and Merckelback [108] state

… to clarify the nature of the atypical symptoms, SVTs are administered and a negative response bias is found, which is explained away by the atypical symptoms. Negative response bias allows for only one conclusion: the patient’s self-report of symptoms and life history can no longer be taken at face value (p. 122).

The problem with a singular conclusion like this is that typically nothing other than the SVT measure is used to independently show response bias and no other medical, emotional or injury issues are controlled [109, 110]. Thus, inferred negative response bias with SVT failure is a common theme and may be a totally appropriate conclusion, but, unless other evidence explicates the role of response bias, a simple below cut-score but above chance SVT performance does not prove response bias.

**Figure 4.**
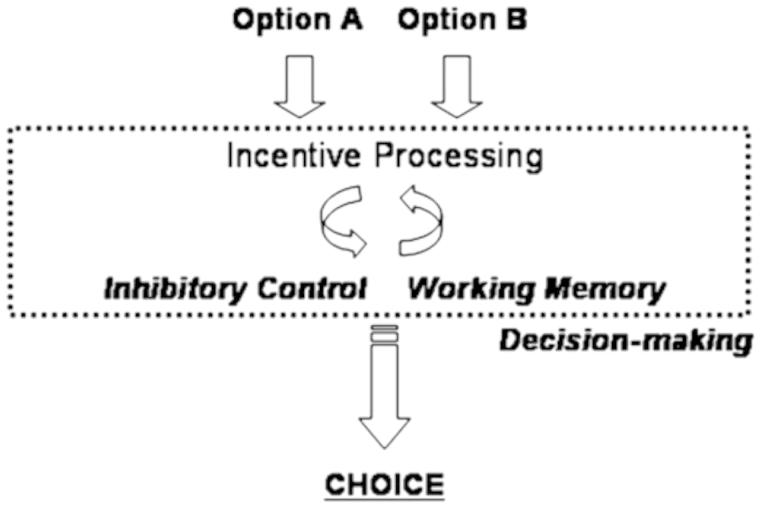
As presented by Geier and Luna [111], a simple model emphasizing the interaction between incentive processing and basic cognitive control abilities in decision-making. Sub-optimal decision-making has been suggested to contribute to risk-taking behaviour. In the Geir and Luna model, immaturities in brain systems supporting how incentives are represented in the brain as well as in specific cognitive control systems like working memory and inhibitory control are proposed to underlie poor decision-making. As pertaining to SVT performance, damage within inhibitory control, working memory and/or incentive processing systems may result in SVT errors.

Furthermore, there is an anatomical basis to response bias, which involves frontotemporolimbic and basal ganglia circuitry [111]. Figure 4 depicts a very simple model proposed by Geier and Luna [111] that shows the interaction of incentive, inhibitory control, working memory and decision-making. In a very coarse sense, inhibitory control is mediated by networks associated with the basal ganglia and frontal executive, whereas the working memory system would be mediated by temporal and default mode networks. Incentive would be mediated by frontotemporolimbic networks. Response bias would be influenced by how these networks interact in producing behaviour. With an underlying neurobiology sub-serving response bias but current SVT interpretation based solely on behavioural response indices, the entire underlying neurobiology of response bias has not been explored.

Returning to the McCauley et al. [76, 77] investigations that showed the influence of incentive on prospective memory function in children, in a ‘high-motivation’ condition (where a dollar was given for remembering a phrase when the prospective cue was given) incentive to increase performance was found to be mediated by white matter integrity within the orbitofrontal, cingulum bundle and uncinate fasciculi along with injury severity. All of these regions are well known for their role in motivated behaviour. On average control participants and those with moderate injury were able to improve their prospective memory performance by 1–2 more phrases recalled in the ‘high motivation’ condition. However, the children with severe TBI and reduced white matter integrity did not change under either incentive condition. This study clearly points to an underlying neurobiology related to the effort to perform, with implications for SVT performance.

Examining the influence of incentive in test performance supports the concept that better ‘effort’ results in better performance [112] and the potential appropriateness of the effort term in understanding SVT performance [113–115]. However, the operational definition problem with the ‘effort’ term in SVT studies is that effort is never directly defined independent of the SVT measure, only inferred by SVT performance. The tautological restrictions of defining poor effort by the criterion variable to identify effort become obvious when there is no other criterion used to define ‘effort’ [10]. Further complicating nomenclature and meaning of labels, some have added additional qualifiers such as ‘good’ or ‘poor’ effort, where ‘poor effort’ becomes synonymous with malingering. The conclusion of malingering is much more than just SVT failure [116].

In reviewing the role of top-down neural control, as shown in Figure 1, the next step for SVT research is to begin to better understand the role of top-down neural systems that may participate in SVT performance. Better understanding the neural basis of SVT performance may ultimately lead to improved operational definitions for validity testing including use of the ‘effort’ term. As Ruff [117] states ‘How such top-down control processes may ultimately be guided by motivational brain systems is a topic of current debate’ (p.88, [118]). To date, there are no SVT studies that comprehensively address these issues using neuroimaging technology.

While below cut-score SVT performance relates to increased symptom endorsement in individuals with mTBI [119, 120], since most neuropsychological tests measure cognitive ability, Larrabee [41] suggests that the term ‘performance validity’ should be used instead of SVT or ‘effort’ or other terms discussed in this section or previously mentioned in this review. Performance validity testing or PVT is probably a much more accurate description, because the cognitive measure is not necessarily evaluating a symptom. Furthermore, some may be accurately reporting symptoms yet inaccurately displaying the true cognitive performance related to the symptom [16].

The importance of the SVT vs. PVT differentiation is borne out in the study by Van Dyke et al. [121] of a large mixed referral veteran sample (*n* = 120). Confirmatory factor analysis was used to determine the best factor model describing the relation between cognitive performance, symptom self-report, performance validity and symptom validity. They concluded that

the strongest and most parsimonious model was a three-factor model in which cognitive performance, performance validity and self-reported symptoms (including both standard and symptom validity measures) were separate factors. The findings suggest failure in one validity domain does not necessarily invalidate the other domain. Thus, performance validity and symptom validity should be evaluated separately (p. 1234).

† Although PVT is the better term to use in reference to any cognitive measure, for the remainder of this review the SVT acronym will be retained.


## SVT measures and the role of neuropsychological testing in classification of neural impairment in mTBI

**Figure 5.**
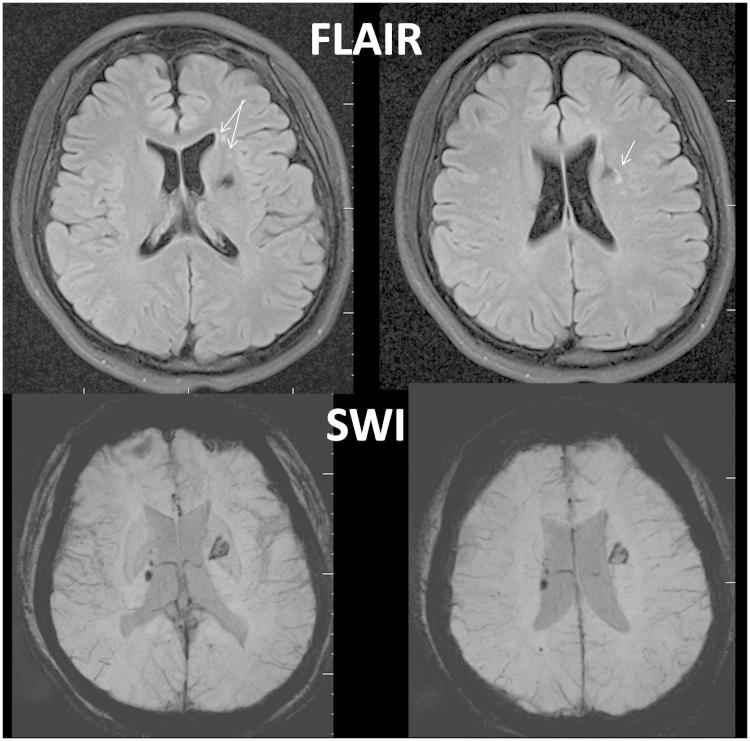
In this patient who sustained a mTBI, MRI studies performed more than 1 year post-injury show areas of hemosiderin deposition as numerous chronic haemorrhagic lesions (multifocal hypointense regions of dark signal). Note, in association with the hemosiderin depositions are scattered white matter hyper-intense signal abnormalities (white arrows).

Prior to contemporary advancements in neuroimaging, neuropsychological assessment techniques were relied on to define the presence of ‘organicity’, mid-20th Century parlance for neural impairment or ‘damage’ [122, 123]. Figure 5 is from a patient who sustained a mTBI with a Glasgow Coma Scale (GCS) of 14 in transit but 15 in the emergency department. CT imaging was positive for intra-parenchymal haemorrhage within the region of the left basal ganglia. Follow-up MRI more than 1 year post-injury, when neuropsychological testing was undertaken, showed the old haemorrhagic lesion within the left lenticular nucleus but also numerous old haemorrhagic foci that were not detected on the day-of-injury CT. Additionally, several white matter hyperintensities were observed in conjunction with the haemorrhagic foci, an indication of traumatically-induced white matter pathology [68]. Note the frontal distribution. On three separate SVT measures this individual scored one item below the cut-point. Does this invalidate anything? It certainly does not invalidate the objective evidence that a significant brain injury had occurred. Given the frontal pathology and basal ganglia damage, is this not an explanation for deficits in attention, drive and cognitive engagement that could result in slightly reduced SVT performance [124, 125]?

Neuroimaging studies, rigorously performed, show a variety of abnormalities and lesion types that may occur in mTBI, including their frontotemporlimbic distribution [68, 126–128]. In the presence of positive neuroimaging findings in mTBI, identification of whether a brain injury has occurred or not is no longer the role of a neuropsychological examination, whether valid or invalid. Recent neuroimaging findings even bring in to question what is the meaning of SVT failure, as described in the above cases shown in Figures 2 and 5 or as reported in the Hetherington et al. [129] study of 25 veterans who sustained mTBI from blast injury. These veterans underwent MRI at 7 Tesla that included MR spectroscopic (MRS) studies. MRS findings have been shown to correlate with traumatic axonal injury in TBI [130] along with TBI sequelae [131]. Hetherington et al. demonstrated that high-field MRS findings suggesting metabolic abnormalities were present in the hippocampus in these veterans, including those who ‘failed’ SVT testing. Could subtle hippocampal pathology disrupt attention, motivation and emotional valence, even for tasks that should be easily performed?

What does this mean for neuropsychological test performance in general and SVT measures specifically? Unknown, but clearly the neuropsychologist is now confronted with major Type 1 vs. Type 2 statistical dilemmas. How would the significance of a mTBI be discounted by a failed SVT measure in the presence of objectively demonstrated hippocampal pathology defined by impaired metabolic functioning? Particularly problematic would be the neuropsychologist who concludes on the grounds of ‘SVT Failure’ that the veteran’s symptoms were malingered, all-the-while knowing the scan findings are abnormal and a possible explanation for why ‘failure’ occurred. Such examples provide alternative explanations to SVT failure [12].

Rienstra et al. [132] examined 170 consecutive patients in a memory clinic where overall only 6.5% failed FC SVT measures. As such, validity of performance is assumed for the majority of these patients, as addressed by the SVT ‘pass’. While hippocampal volume correlated with memory performance in those who passed SVT measures, it was unrelated to memory performance in those who failed FC SVT measures. Does this line of research open up an avenue of examining SVT performance in terms of quantitative neuroimaging? Goodrich-Hunsaker and Hopkins [133] showed that FC SVT performance was right at the cut score in three amnesic patients with anoxic injury who had reduced hippocampal volume. Of interest, in the Hunsaker-Goodrich and Hopkins study, a single additional error and the performance would be marked as ‘failed’ in each of the three individuals examined. More studies like these are needed to better understand SVT outcome.

**Figure 6.**
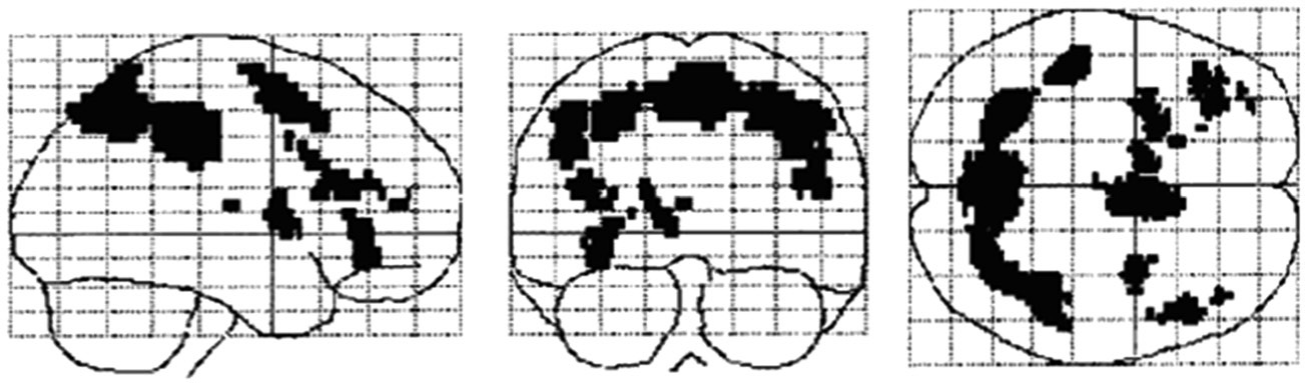
Callicott et al. [137], in an n-back design with non-TBI, typical developed controls manipulated cognitive load and identified the working memory network as shown in the glass brain model. Reproduced with permission from Oxford University Press.

fMRI studies in mTBI that examine task complexity and stimulus load, such as required by n-back designs, show additional cortical recruitment to perform similarly to controls [134, 135]. In other words, if underlying subtle neuropathology is present in mTBI, additional brain regions must be recruited in order to maintain performance at a level similar to the non-injured control [136]. Callicott et al. [137], in an n-back design with non-TBI, typical developed controls manipulated cognitive load and identified the working memory network, as shown in Figure 6. These are in fact the same regions where greater recruitment is necessary for individuals with mTBI to perform comparable to controls [138–140]. This is the default mode network (DMN) critical for attention and working memory (see Figure 1 and the critical role of the DMN in top-down cognitive control of effort) where the Zhou et al. [141] study showed abnormal DMN connectivity associated with mTBI, in patients with otherwise negative MRI. The SVT study in mTBI that examines working memory deficits with subjects that exhibit subtle n-back working memory impairments and more extensive recruitment to maintain ‘normal’ memory performance has not been done. Likewise, using advanced neuroimaging studies like the Zhou et al. [141] study, no SVT study has been done where DMN abnormalities have been identified and examined systematically. Since problems with working memory occur not only in mTBI, but in associated or co-morbid PTSD, anxiety and depression-related disorders with mTBI, could this be a potential explanation why some studies with a military population and these co-morbidities have such high SVT failure rates? Systematic SVT studies examining these issues are needed.

Disruption in DMN integrity may have other implications for SVT performance. WM impairment is present in ADHD [142–145], which is thought to be related to deficits in the central executive [146]. Marshall et al. [147] found a 22% SVT failure rate in 268 adults referred for ADHD assessment. Are any of these failures associated with working memory/DMN impairment? How would one know without an integrated neuropsychological paradigm with either a functional neuroimaging/electrophysiological study of the DMN network? With the combination of problems in error monitoring, DMN functioning and the central executive in TBI [148, 149], plausible explanations potentially exist for SVT errors resulting in below traditional cut-score performance in the individual with a history of mTBI.

## Are there neuropathological conditions that routinely affect performance validity?

**Figure 7.**
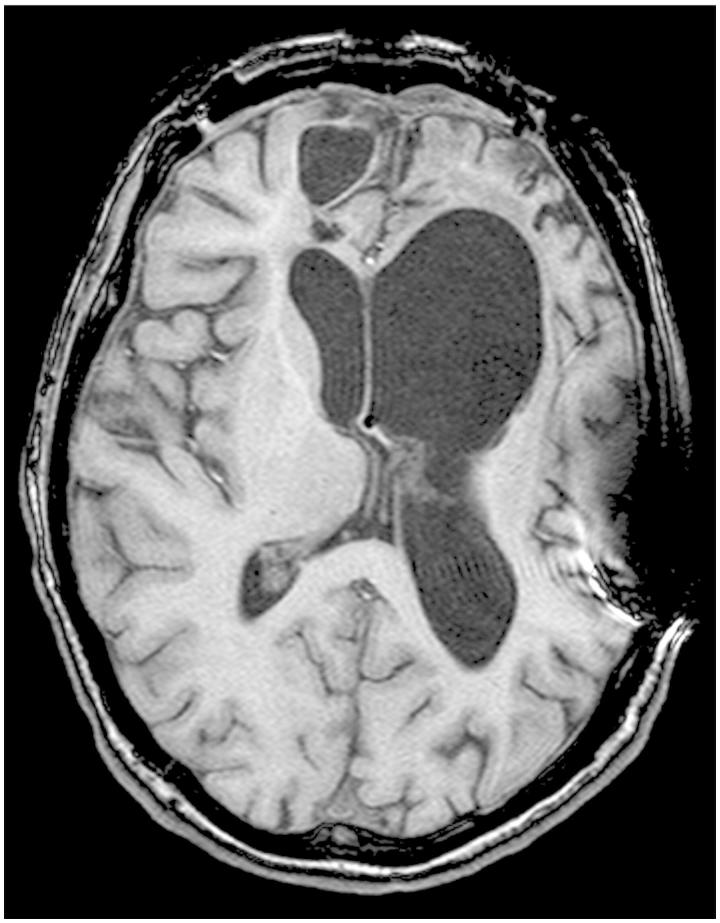
T1 weighted image of extensive damage in severe TBI where the patient either performed without error or only made one error across three SVT measures. The scan is in radiological perspective with left on the viewer’s right. Note that, despite the distinct bifrontal pathology, this patient with severe structural damage had no difficulty passing all SVT measures administered.

Performance validity tests are failed by a variety of neurological conditions directly attributable to the extent and severity of the neurological disorder [132]. Although only a few anecdotal case studies have been published to date showing SVT failures associated with temporal lobectomies and the chronic effects of herpes simplex encephalitis [10], there are also case studies that show patients with hippocampal atrophy and hippocampectomies who readily pass SVTs [133, 150]. Figure 7 shows a patient with severe TBI with massive structural damage, including bi-frontal pathology, yet on multiple SVT measures this patient either performed without error or, at the most, one error.

Understandably, more advanced stages of dementia are associated with SVT failure, as are conditions of mental retardation, illiteracy or restricted educational opportunities and schizophrenia [151–154]. For example, Sieck et al. [155] examined SVT performance in a group of patients with Huntington Disease (HD). The diagnosis was not in dispute and no patients were seeking compensation; however, depending on which SVT was used, somewhere between 8–18% of the sample of 36 HD patients ‘failed’ the SVT measure. Of major interest was that SVT ‘failure’ occurred in those with greater motor and cognitive impairment, thereby suggesting that the disease influenced SVT performance. Basal ganglia play critical roles in drive and motivation—are these factors associated with SVT in HD?

Some suggest a ‘profile analysis’ applied to the SVT task to interpret whether SVT failure may actually be influenced by the disorder being evaluated, especially in cases of dementia [156]. The original idea of a SVT measure for commenting on validity was its brevity and the simplicity of the pass/fail dichotomy, permitting the clinician/researcher to make a quick decision. However, despite their simplicity, memory-based FC measures do test memory function [157] and potentially unique profiles have to be examined for each clinical condition being examined.

## Is there a neurobiology of illness or sickness behaviour that relates to test validity

The concepts of illness or sickness behaviour were presented early in this review. As quoted from Sirri et al. [158],

The concept of illness behaviour was introduced to indicate the ways in which given symptoms may be perceived, evaluated and acted upon at an individual level. Illness behaviour may vary greatly according to illness related, patient-related and doctor-related variables and their complex interactions (p. 74).

A patient who has a legitimate TBI may not only manifest behaviours associated with the brain injury, but so-called ‘illness behaviour’ [159]. While there are a host of pre-injury/illness and psychological variables that underlie illness behaviour [158, 160], inflammation, either systemic or directly within the central nervous system, may mediate aspects of sickness behaviour [161], complicating the expression of illness behaviour as a functional disorder [162]. This becomes a particularly important issue with regards to TBI, because of the vulnerability of white matter in the process of mechanical deformation from head injury and the role that even subtle neuroinflammation from trauma induced pathology may play in the neurobehavioural expression of TBI [163]. The title of Irwin’s [164] review—Inflammation at the intersection of behaviour and somatic symptoms—captures the blurring of the biological with what may otherwise be termed psychogenic.

Functional neuroimaging studies are now illuminating potential neural correlates of what may be considered functional disorder [165, 166]. These studies show frontotemporlimbic differences in activation. What does it mean to have a so-called functional disorder where these brain regions may activate in anomalous ways in the context of performance validity and neuropsychological testing?

Part of illness or sickness behaviour may be a feeling that anything that requires sustained attention is burdensome [167–169]. Indeed, some describe this as “mental fatigue” [170]. In depressed patients with prominent rumination over symptoms, increasing the demands of a memory task results in reduced resource allocation to adequately perform [171]. TBI and especially mTBI has as one of its common symptoms a complaint of mental fatigue (also this symptom is related to mental exertion and headache). Within cognitive neuroscience, mental effort can be operationalized in terms of task duration and complexity [172, 173]. Interestingly, in such paradigms increased effortful retrieval involves complex interactions between medial temporal lobe structures, the hippocampus in particular, and attentional/default mode networks [174]. How these neural systems function in disorders associated with illness behaviour and SVT performance is unknown.

So, does experiencing a distressing symptom result in network disruption necessary for managing mental effort? Could this be a factor in the high SVT failure rate in mTBI? This is theoretically plausible but to date never empirically tested. Is this another potential explanation as to why SVT failure is so high in OEF/OIF military cases [49]?

In some who have sustained a mTBI, research has shown greater cortical recruitment just to maintain normal levels of test performance and Dobryakova et al. [175] have also shown greater recruitment of the basal ganglia and prefrontal cortex is necessary to perform similarly to controls. In those with subjective complaints of fatigue, what does greater recruitment mean in terms of performance validity and mental effort? This is unknown.

There is an extensive literature on neural correlates of attentional bias [176–178]. How attentional biases, regardless of whether functional or biological, may influence mTBI performance during SVT measures has not been systematically examined.

## Practical questions and issues that need answers

Dandachi-FitzGerald et al. [4], in their review of practices and beliefs of neuropsychologists using SVT measures, state the following:

There is little consensus among neuropsychologists on how to instruct patients when they are administered SVTs and how to handle test failure. Our findings indicate that the issues regarding how to administer and communicate the SVT results to patients warrant systematic research (p. 771).

This review re-affirms the numerous limitations in the current understanding of SVT findings.

The typical neuropsychological test battery requires several hours of administration time and currently most tests have no built-in SVT measure, which is why external SVT administration was developed. Even screening measures, comprised of tasks that assess several domains of cognitive functioning, will take 30–60 minutes to administer. So the question has been asked as to how many validity tests should be administered [179, 180]? Does one need to be administered for each domain [181]?

New test development and re-standardization of existing tests are moving toward having embedded measures within the neuropsychological test. Almost all external SVTs utilize some aspect of memory performance, yet the typical comprehensive neuropsychological examination evaluates motor, sensory-perceptual, language, visual-spatial, processing speed and executive function in addition to memory [18]. Given that the external SVT is never part of the actual target test performance, there will always be the question as to validity with external SVTs and individual test performance on non-memory tasks. Given these current limitations, future neuropsychological test development should focus on internal, not external performance validity measures for each measure and not external SVT tasks.

The inter-relationship of one PVT with others and test–re-test variability within current PVT measures has not been extensively examined. How does normal fluctuation in testing relate to PVT findings? The test–re-test reliability estimates reported by Dikmen et al. [50] for the Halstead-Reitan Neuropsychological Test battery ranged from ‘0.70 to low 90s’ (p. 346). What accounts for this variability and is variability just a dimension of performance validity? What are the test–re-test reliability features of PVT measures by diagnostic category of various neurological diseases and disorders? How frequently do patients fail PVT measures during one testing session and not another? Systematic studies comparing different PVT measures and test–re-test variability have never been done. With multiple PVT measures during a neuropsychological battery, is there any practice or order effect of PVT administration and how would this compound test–re-test variability?

## Summary and conclusions

As discussed in this review, the majority of individuals with a history of TBI undergoing neuropsychological testing perform above cut-points on SVT measures. By current practice standards, passing SVT measures is likely the best indicator of generally valid test findings. Likewise, substantially below cut-point performance that nears chance or is at chance signifies invalid test results. Significantly below chance is the *sine qua non* indicator for malingering.

Below the cut-point yet far above chance is where SVT research needs to clarify the meaning of such test findings. Case studies presented in this review show the problems with rigidity in interpretation with traditionally established cut-scores in some cases of TBI. A better understanding of how certain types of neurological or neuropsychiatric or even test conditions may affect SVT performance is needed, especially integrated with what advanced neuroimaging techniques offer in examining these conditions.

## Declaration of interest

No grant funding supported the writing of this review. The author does provide forensic consultation in the course of his clinical work as co-director of the Neuropsychological Research and Clinical Assessment Laboratory.
